# High‐fat diet induced obesity and age influence the telomere shelterin complex and telomerase gene expression in mouse adipose tissue

**DOI:** 10.14814/phy2.14461

**Published:** 2020-06-08

**Authors:** Samuel I. Bloom, Andrei Tuluca, Stephen J. Ives, Thomas H. Reynolds

**Affiliations:** ^1^ Department of Health and Human Physiological Sciences Skidmore College Saratoga Springs NY USA; ^2^ Department of Nutrition and Integrative Physiology University of Utah Salt Lake City UT USA; ^3^ College of Medicine Central Michigan University Mount Pleasant MI USA

**Keywords:** aging, obesity, telomeres

## Abstract

Obesity and aging are linked to inflammation and increased risk of chronic disease. Telomeres are the endcaps of chromosomes that are regulated by telomerase, the enzyme that elongates telomeres, as well as a protein complex known as shelterin. Telomere dysfunction is associated with inflammation, aging, and disease. However, the effect of high‐fat diet (HFD) induced obesity and advancing age on the shelterin complex and telomerase in adipose tissue is unknown. The present study investigated the effects of obesity and aging on C57BL/6J mice adipose tissue mRNA expression of shelterin complex genes. Young (YG) mice (3 mo) were randomly assigned to be fed either a high‐fat diet (YG + HFD; 60% kcal from fat) or a low‐fat diet (YG + LFD; 10% kcal from fat). A subset of mice were aged until 16 months. Body weight and epididymal white adipose tissue (EWAT) weight increased with age or a HFD. There was a trend for increased *Terf2* expression, as expression was increased in HFD + YG by ~47% and aged mice by ~80%. *Pot1b* expression was increased in aged mice by ~35%–60% compared to YG, independent of diet. *mTert*, the gene that codes for the catalytic subunit of telomerase, was significantly elevated in aged mice. Changes in telomere associated gene expression was accompanied by changes in expression of inflammatory markers *Mcp1* and *Tnfα*. These findings suggest obesity and age impact expression of shelterin complex and telomerase related genes in adipose, perhaps altering telomere function in adipose tissue thereby increasing inflammation and risk of chronic disease.

## Graphical abstract

We investigated whether high‐fat diet (HFD) induced obesity or advancing age affect gene expression of the telomere shelterin complex and telomerase associated genes, as well as markers of cellular senescence and inflammation. Our findings suggest that obesity and age impact the expression of shelterin complex and telomerase related genes in adipose tissue, perhaps altering telomere function and, thereby, increasing inflammation and risk of chronic disease.

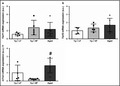

## 1 INTRODUCTION

In the United States, the prevalence of obesity is increasing (Hales, Carroll, Fryar, & Ogden, 2017) alongside the number of elderly individuals (He, Goodking, & Kowal, 2016). Both obesity and aging are independent risk factors for many chronic diseases, including type 2 diabetes (Barnes, 2011; Lindstrom & Tuomilehto, 2003), cardiovascular disease (Dhingra & Vasan, 2012; Sowers, 2003), and some forms of cancer (De Pergola & Silvestris, 2013; U.S. Cancer Statistics Working Group, 2013). Therefore, it is probable that similar physiological processes are central to the increased risk of disease found with aging and obesity. However, many questions about how aging and obesity lead to disease remain poorly resolved. In addition to physical inactivity and other lifestyle factors, a high‐fat diet (HFD) is associated with obesity (Golay & Bobbioni, 1997). Obesity and aging are characterized by disruption of adipose tissue homeostasis (Goossens, 2008; Kirkland, Tchkonia, Pirtskhalava, Han, & Karagiannides, 2002); accordingly, understanding how obesity and advanced age influence adipose tissue may delineate mechanisms responsible for the increased risk of disease associated with obesity and advancing age.

Adipose tissue is a highly active organ involved in various physiological processes including energy storage, metabolism, and the secretion of hormones and signaling molecules (Frayn, 2002; Goossens, 2008). It has been established that dysfunctional adipose tissue is involved in the inflammatory response, (Trayhurn & Wood, 2004) the development of insulin resistance (Goossens, 2008), and both aging and obesity can disrupt normal adipose tissue function (Goossens, 2008; Kirkland et al., 2002). Disruption of adipose tissue homeostasis may be caused, in part, by an increase in oxidative stress (Zhang et al., 2011). According to the free‐radical theory of aging, oxidative stress increases with age (Harman, 1992) leading to damage to DNA, lipids, and proteins – changes which affect physiological function by promoting inflammation and altering cell cycle control (Liguori et al., 2018). Likewise, a HFD has been shown to increase oxidative stress (Du et al., 2012) and inflammation (van der Heijden et al., 2015). Adipose tissue dysfunction and the concomitant diseases may therefore be caused by structural and functional damage at the cellular and molecular level.

Telomeres, the end caps of eukaryotic chromosomes, are made of hexanucleotide TTAGGG tandem repeats that are associated with a protein complex known as shelterin. Shelterin proteins are involved in structural formation and protection of telomeres, and in conjunction with the ribonucleoprotein reverse transcriptase known as telomerase, modulate telomere length (de Lange, 2005). Shelterin proteins regulate the structure of telomeric DNA, forming what is known as a t‐loop to cap telomeres (de Lange, 2009). Telomere capping prevents recognition of chromosome ends as damaged DNA, thereby preventing the initiation of DNA damage response pathways that lead to cellular senescence or apoptosis (Takai, Smogorzewska, & de Lange, 2003). Dysfunctional telomeres have been linked to advancing age (Morgan et al., 2013), and a variety of diseases (Kong, Lee, & Wang, 2013) including cardiovascular disease (Liu, Bloom, & Donato, 2019; Morgan et al., 2014) and cancer (Shammas, 2011). Thus, it is important to understand telomere dynamics (e.g., telomere uncapping or altered shelterin complex) and the effects of various lifestyle factors, which may occur independent of telomere length (Morgan et al., 2013; Walker et al., 2016). The shelterin complex is comprised of protection of telomeres 1 (POT1) in humans, and its two paralogs, POT1a and POT1b in mice, which function to prevent the damage response along with telomere‐repeat binding factor 1 (TRF1) and 2 (TRF2) (Hockemeyer, Daniels, Takai, & de Lange, 2006; de Lange, 2009; Sfeir et al., 2009). Despite these telomere maintenance and damage prevention mechanisms, it is known that oxidative stress can damage telomeres (Wang et al., 2010; von Zglinicki, 2002), and that inflammation is closely associated with this process (Zhang et al., 2016).

Both advancing age and obesity have been shown to increase oxidative stress (Liguori et al., 2018; Marseglia et al., 2014) and inflammation (Ellulu, Patimah, Khaza'ai, Rahmat, & Abed, 2017; Franceschi & Campisi, 2014), which may cause telomere dysfunction (Wang et al., 2010; Zhang et al., 2016) and cellular senescence (Liu et al., 2019). Furthermore, DNA damage signaling disrupts adipose tissue homeostasis and systemic metabolism (Vergoni et al., 2016), and telomeres are likely targets of DNA damage that can cause cellular senescence (Liu et al., 2019). Therefore, it is logical to examine the effects HFD induced obesity and age on regulators of telomere homeostasis. Additionally, it is important to examine markers of inflammation and cellular senescence to determine if these processes are accompanied by changes in genes that regulate telomere dynamics in adipose tissue. Investigating the relationship between HFD induced obesity, aging and telomere dynamics could help to determine if a HFD accelerates biological aging of adipose tissue. The impact of HFD induced obesity and aging on shelterin proteins that regulate and protect telomeric DNA have yet to be examined in adipose tissue. We hypothesized that both a HFD and aging would induce changes in shelterin protein gene expression indicative of telomere dysfunction, in conjunction with increased expression of pro‐inflammatory cytokines and markers of cellular senescence.

## 2 METHODS

### 2.1 Ethical approval

All animal studies guidelines set forth by the National Research Council's Guide for Care and Use of Laboratory Animals (Institute of Laboratory Animal Resources, Commission on Life Sciences, 2011) were followed, and experimental protocols were approved by the Skidmore College Institutional Animal Care and use Committee (Protocol #123, approved 02/17/2014). The procedures were conducted in accordance with recent guidance provided by the journal (Grundy, 2015).

### 2.2 Animals and diet

15 male C57BL/6J mice were purchased from Jackson Laboratory (Bar Harbor, ME). Young (YG) mice were randomly assigned to be fed either a high‐fat diet (YG + HFD; *n* = 5; 60% kcal from fat) or a low‐fat diet (YG + LFD; *n* = 4; 10% kcal from fat) for 3 months (Test Diets). A subset of mice were fed a low‐fat diet (*n* = 6; 10% kcal from fat) and aged until 16 months (Test Diets). Animals were housed at the Skidmore College animal facility on a 12:12 light:dark cycle and had access to food and water ad libitum.

### 2.3 Tissue harvest

Mice were anesthetized with a 1:1:1 mixture of promace, ketamine hydrochloride, and xylazine by intraperitoneal injection (1.5 ml/Kg). After the removal of EWAT, unconscious mice were euthanized by cervical dislocation. Dissected EWAT was placed in liquid nitrogen, and then stored at −80°C until analysis.

### 2.4 RNA extraction and gene expression quantification

RNA was extracted from EWAT using an RNA extraction kit specific to lipid rich tissues (QIAGEN). Total nucleic acid content was quantified using a spectrophotometer (NanoDrop, Thermo Scientific), and values were used to obtain 1 ug of RNA. RNA was reverse transcribed into cDNA using an Ambion RETROscript Kit (Austin, TX). Quantitative polymerase chain reaction (qPCR) was performed using TaqMan Gene Expression Assays and a StepOne Plus Real‐Time PCR System (Applied Biosystems, Foster City, Ca). The delta‐delta cycle threshold (ΔΔCT) method was used to determine relative quantification.

### 2.5 Statistical analysis

A one‐way analysis of variance (ANOVA) was used to detect statistical differences between the YG + LFD, YG + HFD, and aged groups respectively. A Tukey HSD post‐hoc analysis was used to locate the significance. Values that were two standard deviations away from the mean were considered as outliers and were not included in analysis. To assess the correlation between body weight, EWAT, and mRNA expression, a bivariate correlation analysis was performed. Data are presented as mean ± *SD*, and the level of statistical significance was set at *p* < .05.

## 3 RESULTS

### 3.1 Body weight and adipose tissue mass

**FIGURE 1:**
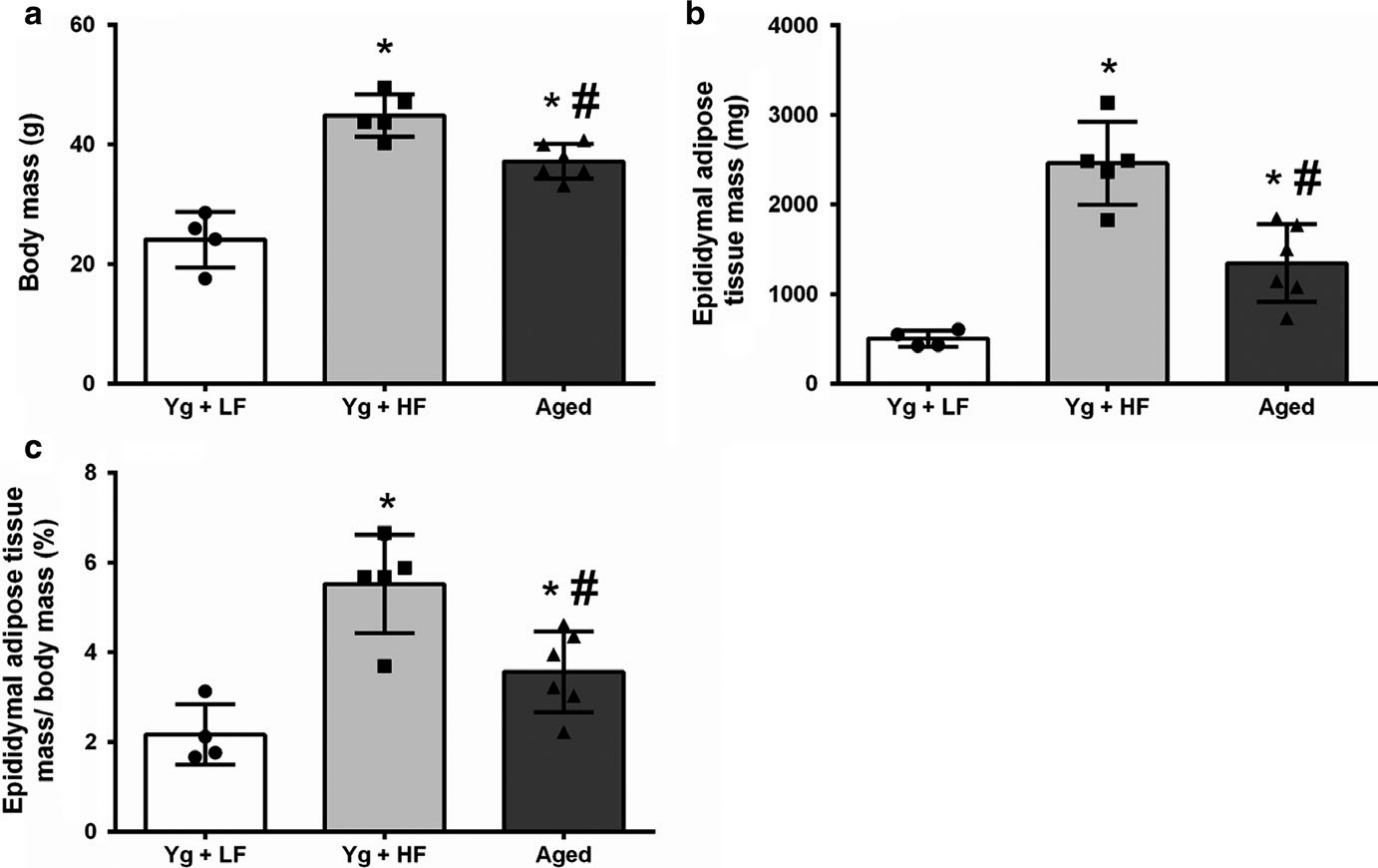
High‐fat diet induced obesity and aging increase body weight and EWAT weight. Young mice were fed either a low‐fat diet (10% kcal from fat) or a high‐fat diet (60% kcal from fat). Aged mice were fed a low fat diet (10% kcal from fat). (a) Body weight and (b) epididymal white adipose tissue (EWAT) weight for all groups. (c) EWAT weight as percentage of body mass. Young high‐fat diet, YG + HFD, *n* = 5; young low fat diet, YG + LFD, *n* = 4; old mice, aged, *n* = 6. *Indicates statistically significant difference from YG + LFD (*p* < .05). ^#^Indicates statistically significant difference from YG + HFD (*p* < .05). Data are means ± *SD*

To determine if a high‐fat diet or aging induce obesity, body weight, and EWAT weight were assessed at the end of our study. As expected, YG mice fed a high‐fat diet (YG + HFD) were significantly heavier than aged (*p* = .011) and young mice fed a low‐fat diet (YG + LFD, *p* = .000) while aged mice weighed more than YG + LFD (Figure 1a; *p* = .000). YG + HFD mice had significantly greater absolute and relative (to body mass) EWAT weight compared to YG + LFD mice (Figure 1b and c; *p* = .000), and aged mice (*p* = .001), while aged mice had significantly higher absolute and relative EWAT weight compared to YG + LFD (Figure 1b and c; *p* = .015).

### 3.2 Adipose tissue shelterin complex and telomerase‐related gene expression

**FIGURE 2:**
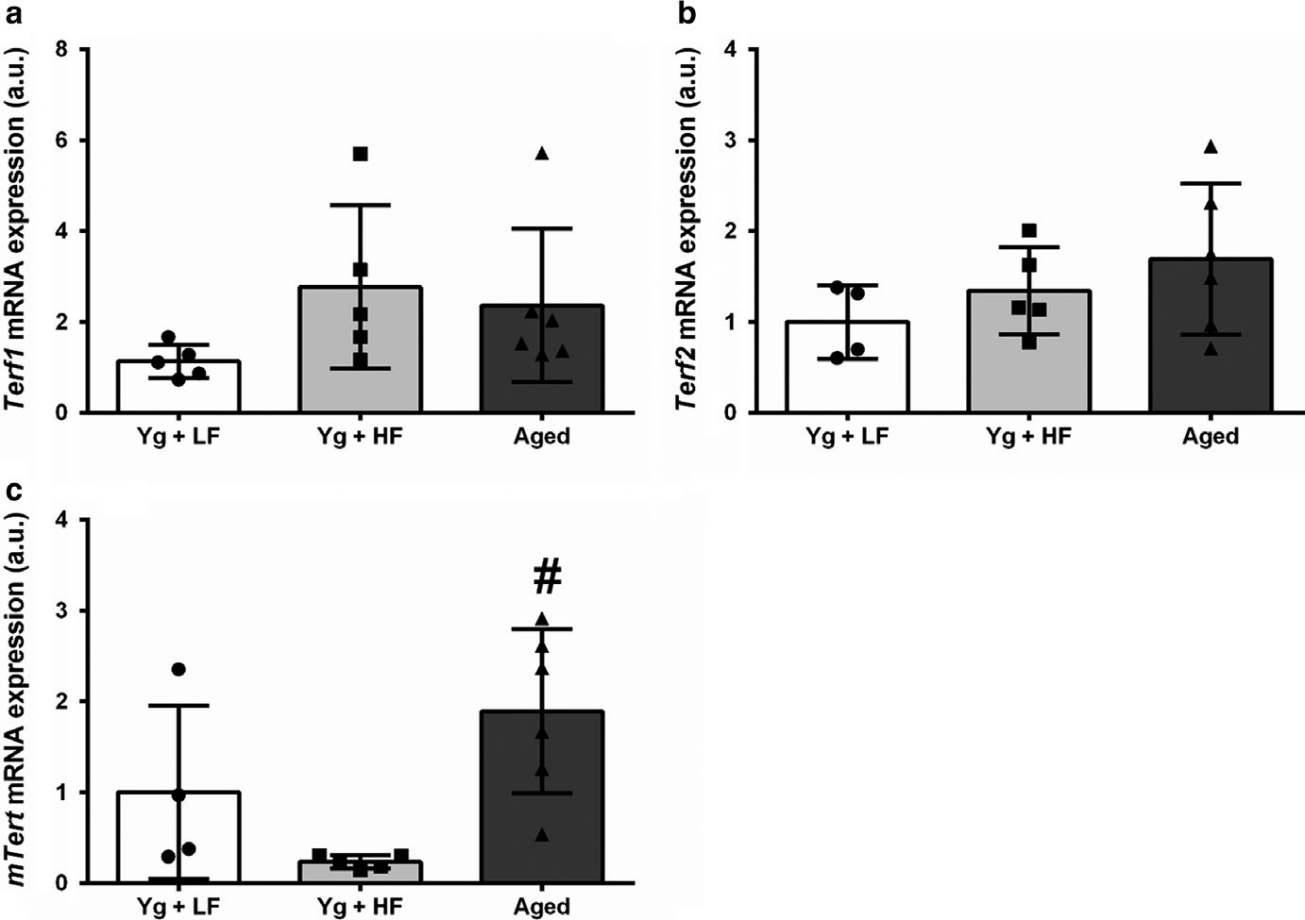
High‐fat diet induced obesity and aging influence adipose tissue gene expression of shelterin protein *Terf1*, *Terf2*, and *mTert*. Young mice were fed either a low‐fat diet (10% kcal from fat) or a high‐fat diet (60% kcal from fat). Aged mice were fed a low fat diet (10% kcal from fat). (a) Telomere‐repeat binding factor 1 (*Terf1*) mRNA expression. (b) Telomere‐repeat binding factor 2 (*Terf2*) mRNA expression. (c) *mTert* mRNA expression. Young high‐fat diet, YG + HFD, *n* = 5; young low fat diet, YG + LFD, *n* = 4; old mice, aged, *n* = 6. *Indicates statistically significant difference from YG + LFD (*p* < .05). ^#^Indicates statistically significant difference from YG + HFD (*p* < .05). Data are means ± *SD*

**FIGURE 3:**
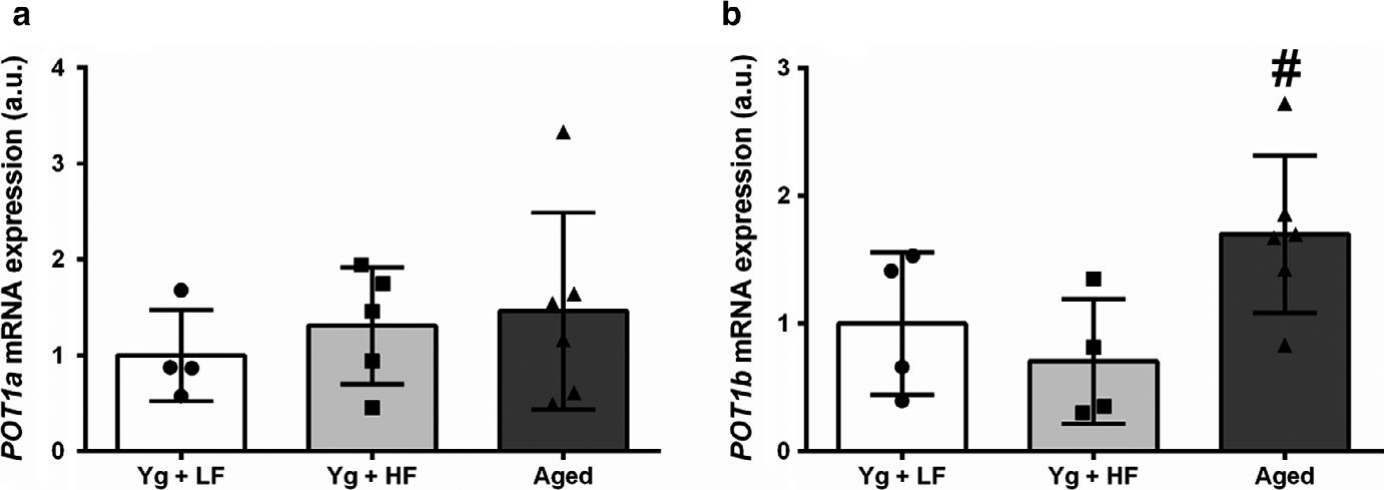
High‐fat diet induced obesity and aging influence adipose tissue gene expression of *POT1a* and *POT1b*. Young mice were fed either a low‐fat diet (10% kcal from fat) a high‐fat diet (60% kcal from fat). Aged mice were fed a low‐fat diet (10% kcal from fat). (a) Protection of telomeres 1A (POT1A) mRNA expression (b) Protection of telomeres 1B (POT1B) mRNA expression; Young high‐fat diet, YG + HFD, *n* = 5; young low‐fat diet, YG + LFD, *n* = 4; old mice, AGED, *n* = 6. ^#^Indicates statistically significant difference from YG + HFD (*p* < .05). Data are means ± *SD*

To investigate the effects of a high‐fat diet and aging on telomere dynamics, we assessed gene expression of shelterin complex proteins that regulate telomere replication, protection, and length. There were no differences in adipose tissue mRNA expression of *Terf1* between groups (Figure 2a; *p* = .233). Aged mice displayed a trend for more *Terf2* mRNA expression than YG + LFD mice (Figure 2b; *p* = .129). There were no significant differences between groups for mRNA expression of *POT1a* (Figure 3a; *p* = .668). Aged mice had significantly greater mRNA expression of *POT1b* compared to YG + HFD (Figure 3b; *p* = .049) and a trend for greater expression compared to YG + LFD mice (*p* = .181). *mTert*, the gene that codes for the catalytic subunit of the enzyme telomerase, was significantly higher in aged compared to the YG + HFD mice (Figure 2c; *p* = .009). To determine if these changes in adipose tissue mRNA expression were associated with changes in body weight or EWAT weight, we performed a bivariate correlation analysis. *mTert* expression tended to be correlated with EWAT weight (*r* = −.502, *p* = .056).

### 3.3 Adipose tissue inflammation and senescence

**FIGURE 4:**
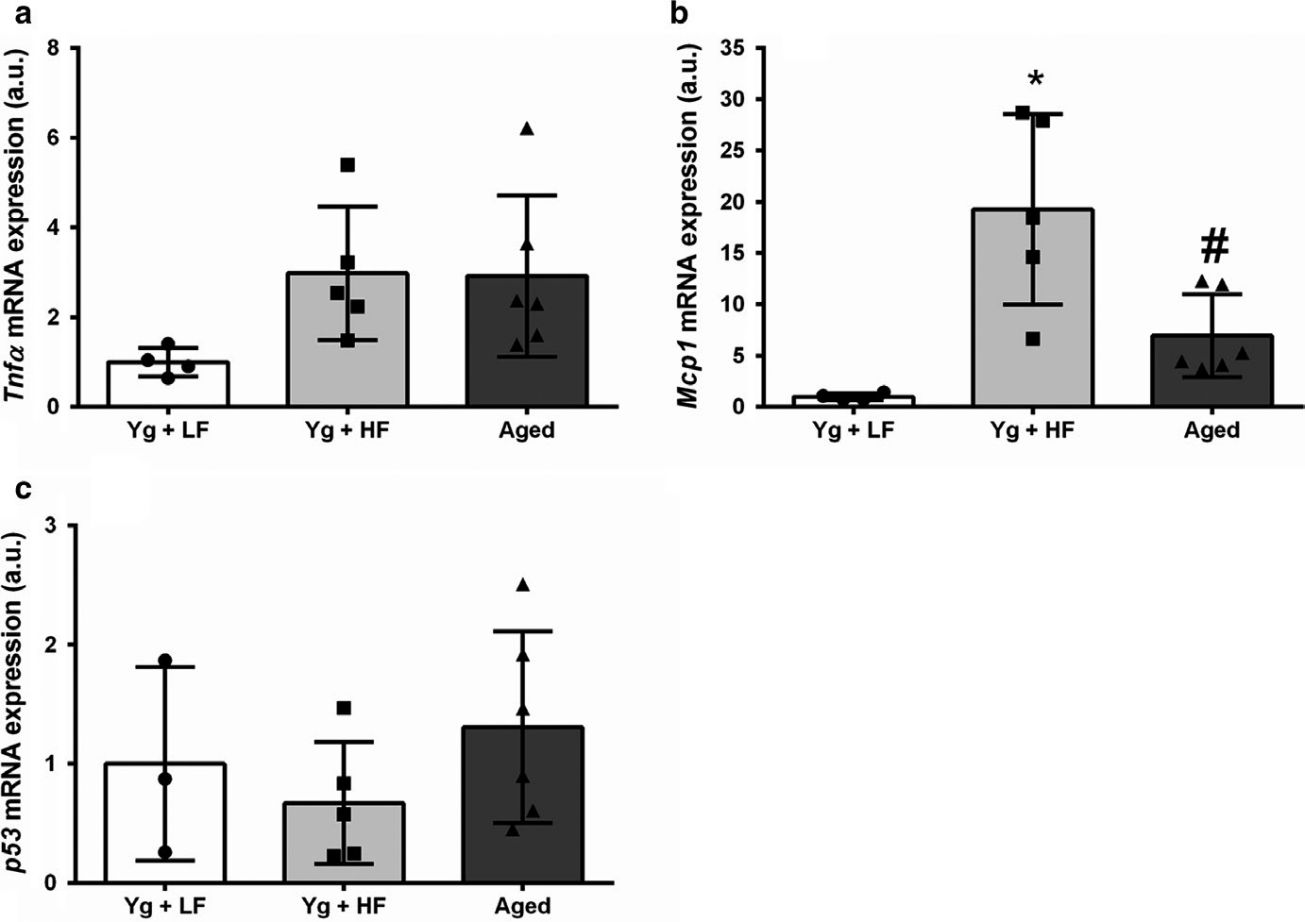
High‐fat diet induced obesity and aging influence gene expression of *Tnfα*, *Mcp1*, and *p53*. Young mice were fed either a low‐fat diet (10% kcal from fat) or a high‐fat diet (58% kcal from fat). Aged mice were fed a low‐fat diet (10% kcal from fat). (a) Tumor necrosis factor α (*Tnfα*) mRNA expression. (b) Monocyte Chemoattractant Protein 1 mRNA expression (MCP1). (c) *p53* mRNA expression; Young high‐fat diet, YG + HFD, *n* = 5; young low‐fat diet, YG + LFD, *n* = 4; old mice, aged, *n* = 6. *Indicates statistically significant difference from YG + LFD (*p* < .05). *Indicates statistically significant difference from YG + LFD (*p* < .05). ^#^Indicates statistically significant difference from YG + HFD (*p* < .05 Data are means ± *SD*

Next, we evaluated the effects of a HFD and age on markers of the inflammatory response. Specifically, we looked at gene expression of monocyte chemoattractant protein 1 (*Mcp*1) and tumor necrosis factor α (*Tnfα*). Adipose tissue *mcp1* mRNA expression significantly elevated in the YG + HFD group compared to the YG + LFD (Figure 4b; *p* = .002) and aged groups (*p* = .013). There was a trend for elevated *Tnfα* in YG + HFD and aged mice versus YG + LFD mice (Figure 4a; *p* = .113). We also assessed gene expression of the tumor suppressor protein *p53*, which is involved in the DNA damage response and cellular senescence. There was no significant difference between groups for *p53* mRNA expression (Figure 4c; *p* = .369). To determine if these changes in adipose tissue mRNA expression were associated with changes in body weight or EWAT weight, we performed a bivariate correlation analysis. *Tnfα* mRNA expression was significantly correlated to body weight (*r* = .524, *p* = .045) and *Mcp1* mRNA expression was significantly correlated to body weight (*r* = .683, *p* = .005) and eWAT weight (*r* = .861, *p* = .000).

## 4 DISCUSSION

The present study was the first to demonstrate the effects of HFD induced obesity and aging on the telomere shelterin complex and telomerase gene expression in mouse adipose tissue. We demonstrate that adipose tissue gene expression of the shelterin protein *Terf2* tends to be elevated in adipose tissue of aged and HFD fed mice. Aged mice had significantly greater mRNA expression of *POT1b* compared to YG + HFD. Furthermore, HFD induced obesity decreased *mTert* expression, while age tended to increase its expression, changes which were correlated with body weight and tended to be correlated to EWAT weight. *Mcp1*, a chemokine that regulates immune cell infiltration and a marker of inflammation, was elevated in HFD mice, and its expression was correlated with body weight and EWAT weight. Therefore, both HFD induced obesity and age significantly influence expression of genes involved in the regulation of telomere dynamics in adipose tissue, changes which may ultimately result in increased inflammation.

### 4.1 Shelterin regulation of telomeres

In the present study, it was demonstrated that aging tends to increase *Terf2* gene expression. Changes in *Terf2* expression may represent the deleterious effects of age and obesity on shelterin regulation of telomeres in adipose tissue, or demonstrate a compensatory mechanism, as TRF2 plays a role in double strand break repair (Bradshaw, Stavropoulos, & Meyn, 2005; Mao, Seluanov, Jiang, & Gorbunova, 2007). TRF2 is essential for maintenance of the telomere t‐loop structure that prevents recognition of telomeric DNA by DNA repair machinery which would lead to chromosome fusions and senescence (van Steensel, Smogorzewska, & de Lange, 1998). Thus, in mice, genetic reduction of *Terf2* results in an accelerated aging phenotype in mouse arteries (Morgan et al., 2019) and a rapid DNA damage response and cell death in skin (Martinez, Ferrara‐Romeo, Flores, & Blasco, 2014). Contrary with this, *Terf2* overexpression leads to an accelerated aging phenotype and cancer in mice, and is increased in some types of human cancer (Matsutani et al., 2001; Munoz, Blanco, Flores, & Blasco, 2005; Oh, Kim, Park, & Park, 2005). These findings indicate that regulation of *Terf2* is critical for maintaining cell and tissue homeostasis. Accordingly, the findings of the present study may demonstrate that aging disrupts the regulation of *Terf2* in adipose tissue. The consequences of these changes are unknown and warrant further investigation.

POT1, and its paralogs *POT1a* and *POT1b* in mice, serve to protect and regulate telomeric DNA (Hockemeyer et al., 2006). Previous research has demonstrated lifestyle interventions, such as exercise, induce tissue specific alterations in promoting gene expression of *Pot1a* and *Pot1b* (Ludlow, Gratidao, Ludlow, Spangenburg, & Roth, 2017; Ludlow et al., 2012). Furthermore, genetic reduction of *Pot1b* in mice results in reduced body size and cell death in highly mitotic tissues (He et al., 2009). *Pot1b* regulates the length of the single stranded and, in conjunction with *Pot1a,* contributes to preventing DNA damage response activation (Hockemeyer et al., 2006). In the present study, adipose tissue *Pot1b* gene expression increased in aged mice compared to YG + LFD and YG + HFD (Figure 3b). These findings suggest that advancing age, but not body weight or EWAT weight per se, lead to increased *Pot1b* expression and supports the notion that *Pot1a* and *Pot1b* are likely regulated in a tissue specific manner. However, the age associated increase in expression *Pot1b* could be compensatory in nature, or specific to the experimental model employed.

### 4.2 HFD induced obesity, aging and *mTert*

Obesity is associated with increased expansion of visceral adipose tissue depots (Sun, Kusminski, & Scherer, 2011). In humans, most somatic cells do not express appreciable levels of *TERT*, (Cong, Wright, & Shay, 2002) the catalytic subunit of the enzyme telomerase that elongates telomeres. However, *Tert* mRNA expression has been associated with tumorigenesis, and significant telomerase activity is found in a vast majority of human tumor cells (Cong et al., 2002), highlighting a role for *mTert* in highly mitotic tissues. Contrary to this, adult mice are thought to maintain telomerase expression in somatic tissues (Prowse & Greider, 1995). Interestingly, in the present study aged mice displayed significant upregulation of *mTert* compared to young mice fed a HFD and a trend for increased *mTert* compared to YG + LFD mice (Figure 2). While *mTert* gene expression is not necessarily indicative of telomerase activity, the finding that *mTert* regulation differs between aging and HFD induced obesity warrants further investigation into the regulation of telomerase in mice and might have implications for more effective use of mice as a model to explore the dynamics of telomerase more typical of humans.

### 4.3 HFD induced obesity, aging, and adipose tissue inflammation

HFD induced obesity and aging are associated with adipose tissue dysfunction, including an increase in expression of pro‐inflammatory cytokines which contributes to the pathogenesis of insulin resistance (Goossens, 2008; Mau & Yung, 2018). In the present study, we evaluated gene expression in epidydimal adipose tissue. Recent evidence suggests that diet induced obesity results in the greatest senescence burden in this adipose tissue depot when compared to other tissue types and adipose depots (Palmer et al., 2019). Furthermore, advancing age results in significant senescent cell accumulation in epididymal adipose (Yousefzadeh et al., 2020). Accordingly, it is logical to investigate the dynamics of shelterin complex gene expression, as the shelterin complex plays a key role in preventing cellular senescence and the associated inflammatory phenotype (Liu et al., 2019). Furthermore, mice with genetically reduced telomerase expression, which develop critically short telomeres after several generations, display elevated adipose tissue senescence burden accompanied by increased gene expression of *Mcp1* and *Tnfα* (Minamino et al., 2009). The present study builds upon these findings by demonstrating that diet induced obesity and advancing age results in altered expression of the genes that regulate telomere dynamics and telomerase, changes accompanied by increased *Mcp1* and *Tnfα* expression. In adipose tissue, *Mcp1* expression increases with high‐fat diet induced obesity (Kanda et al., 2006) and aging (Lumeng et al., 2011), and *Mcp1* overexpression leads to insulin resistance in mice (Kanda et al., 2006). Similarly, adipose tissue expression of *Tnfα* is elevated in obesity (Hotamisligil, Shargill, & Spiegelman, 1993) and aging (Lumeng et al., 2011), and inhibition of *Tnfα* improves insulin sensitivity. These data, alongside those of the present study, indicate that changes in the regulation of telomere associated genes occur in response to stimuli that cause inflammation. Telomere dysfunction is associated cellular senescence and a proinflammatory state, and thus the findings of the present study warrant further investigation into the relationship between telomere dynamics and inflammation in adipose tissue.

### 4.4 Experimental considerations

The present study demonstrates that HFD induced obesity and age alter expression of genes in involved in the regulation of telomeres, namely, *Terf2*, *Pot1b,* and *mTert*. However, limitations to this study include that our study only examined shelterin components and telomerase at the mRNA level, rather than at the protein level. Therefore, it is important to recognize that post transcriptional or translational modifications to these gene transcripts may ultimately affect the levels of proteins that are involved in the regulation of telomeric DNA. However, previous research has demonstrated that reduction in shelterin gene expression is sufficient to induce DNA damage and enhance cancer formation (Hartmann et al., 2016). Work in humans has also documented that changes in telomere uncapping and subsequent activation of senescence pathways can occur independent of changes in telomere length (Morgan et al., 2013; Walker et al., 2016). Though, we acknowledge that measurements of telomere length and protein expression would make our current study more comprehensive, we believe that the findings of our study are the first to demonstrate the effects of diet‐induced obesity and aging on adipose tissue regulation of genes involved in telomere dynamics and thus provide novel insight as well as a detailed roadmap for future studies. One additional factor future studies might consider is the exploration of possible associations of altered telomere dynamics with glucose tolerance and insulin sensitivity, as three months HFD induces insulin resistance in C57BL/6J young mice (Liu et al., 2015) and we (Reynolds et al., 2019) and others (Oh et al., 2016) have documented that changes might begin at 16 months, but are more pronounced at 20 months of age.

## 5 CONCLUSION

The main novel findings of the present study include that HFD induced obesity and aging alter adipose tissue regulation of telomeres. Furthermore, aged mice displayed upregulation of adipose tissue *Terf2* and *Pot1b*. Finally, adipose tissue *mTert* mRNA expression was upregulated in aged mice. Concurrent to changes in genes that regulate telomere dynamics, HFD induced obesity and aging increase adipose tissue inflammation. These findings warrant further studies examining the regulation of shelterin and telomeres in adipose tissue, and the relationship between telomere dynamics and inflammation.

## CONFLICT OF INTEREST

None declared.

## AUTHOR CONTRIBUTIONS

S.I.B., S.J.I., and T.H.R. contributed to the conception and design of the work. S.I.B., A.T., and T.H.R. carried out the data acquisition. S.I.B., S.J.I., and T.H.R. performed the analysis and interpretation of data for the work. All authors contributed to drafting of the work and revising it critically for important intellectual content. All authors approved the final version of the manuscript and agree to be accountable for all aspects of the work in ensuring that questions related to the accuracy or integrity of any part of the work are appropriately investigated and resolved. All persons designated as authors qualify for authorship, and all those who qualify for authorship are listed.

## References

[phy214461-bib-0001] Barnes, A. S. (2011). The epidemic of obesity and diabetes: Trends and treatments. Texas Heart Institute Journal, 38(2), 142–144. 21494521 PMC3066828

[phy214461-bib-0002] Bradshaw, P. S. , Stavropoulos, D. J. , & Meyn, M. S. (2005). Human telomeric protein TRF2 associates with genomic double‐strand breaks as an early response to DNA damage. Nature Genetics, 37(2), 193–197. 10.1038/ng1506 15665826

[phy214461-bib-0003] Cong, Y. S. , Wright, W. E. , & Shay, J. W. (2002). Human telomerase and its regulation. Microbiology and Molecular Biology Reviews, 66(3), 407–425. 10.1128/MMBR.66.3.407-425.2002 12208997 PMC120798

[phy214461-bib-0004] de Lange, T. (2005). Shelterin: The protein complex that shapes and safeguards human telomeres. Genes & Development, 19(18), 2100–2110. 10.1101/gad.1346005 16166375

[phy214461-bib-0005] de Lange, T. (2009). How telomeres solve the end‐protection problem. Science, 326(5955), 948–952. 10.1126/science.1170633 19965504 PMC2819049

[phy214461-bib-0006] De Pergola, G. , & Silvestris, F. (2013). Obesity as a major risk factor for cancer. Journal of Obesity, 2013, 291546 10.1155/2013/291546 24073332 PMC3773450

[phy214461-bib-0007] Dhingra, R. , & Vasan, R. S. (2012). Age as a risk factor. Medical Clinics of North America, 96(1), 87–91. 10.1016/j.mcna.2011.11.003 22391253 PMC3297980

[phy214461-bib-0008] Du, Z. , Yang, Y. , Hu, Y. , Sun, Y. U. , Zhang, S. , Peng, W. , … Kong, W. (2012). A long‐term high‐fat diet increases oxidative stress, mitochondrial damage and apoptosis in the inner ear of D‐galactose‐induced aging rats. Hearing Research, 287(1–2), 15–24. 10.1016/j.heares.2012.04.012 22543089

[phy214461-bib-0009] Ellulu, M. S. , Patimah, I. , Khaza'ai, H. , Rahmat, A. , & Abed, Y. (2017). Obesity and inflammation: The linking mechanism and the complications. Archives of Medical Science, 13(4), 851–863. 10.5114/aoms.2016.58928 28721154 PMC5507106

[phy214461-bib-0010] Franceschi, C. , & Campisi, J. (2014). Chronic inflammation (inflammaging) and its potential contribution to age‐associated diseases. Journals of Gerontology Series A, Biological Sciences and Medical Sciences, 69(Suppl 1), S4–S9. 10.1093/gerona/glu057 24833586

[phy214461-bib-0011] Frayn, K. N. (2002). Adipose tissue as a buffer for daily lipid flux. Diabetologia, 45(9), 1201–1210. 10.1007/s00125-002-0873-y 12242452

[phy214461-bib-0012] Golay, A. , & Bobbioni, E. (1997). The role of dietary fat in obesity. International Journal of Obesity and Related Metabolic Disorders, 21(Suppl 3), S2–11. 9225171

[phy214461-bib-0013] Goossens, G. H. (2008). The role of adipose tissue dysfunction in the pathogenesis of obesity‐related insulin resistance. Physiology & Behavior, 94(2), 206–218. 10.1016/j.physbeh.2007.10.010 18037457

[phy214461-bib-0015] Grundy, D. (2015). Principles and standards for reporting animal experiments in The Journal of Physiology and Experimental Physiology. Journal of Physiology, 593(12), 2547–2549. 10.1113/jp270818 26095019 PMC4500341

[phy214461-bib-0016] Hales, C. M. , Carroll, M. D. , Fryar, C. D. , & Ogden, C. L. (2017). Prevalence of obesity among adults and youth: United States, 2015–2016. NCHS data brief, no 288. Hyattsville, MD: National Center for Health Statistics. 29155689

[phy214461-bib-0017] Harman, D. (1992). Free radical theory of aging. Mutation Research, 275(3–6), 257–266. 10.1016/0921-8734(92)90030-s 1383768

[phy214461-bib-0018] Hartmann, K. , Illing, A. , Leithäuser, F. , Baisantry, A. , Quintanilla‐Martinez, L. , & Rudolph, K. L. (2016). Gene dosage reductions of Trf1 and/or Tin2 induce telomere DNA damage and lymphoma formation in aging mice. Leukemia, 30(3), 749–753. 10.1038/leu.2015.173 26135248 PMC4777776

[phy214461-bib-0019] He, H. , Wang, Y. , Guo, X. , Ramchandani, S. , Ma, J. , Shen, M.‐F. , … Chang, S. (2009). Pot1b deletion and telomerase haploinsufficiency in mice initiate an ATR‐dependent DNA damage response and elicit phenotypes resembling dyskeratosis congenita. Molecular and Cellular Biology, 29(1), 229–240. 10.1128/mcb.01400-08 18936156 PMC2612488

[phy214461-bib-0020] He, W. , Goodking, D. , & Kowal, P. (2016). An aging world: 2015. Washington, DC: Government Publishing Office.

[phy214461-bib-0021] Hockemeyer, D. , Daniels, J. P. , Takai, H. , & de Lange, T. (2006). Recent expansion of the telomeric complex in rodents: Two distinct POT1 proteins protect mouse telomeres. Cell, 126(1), 63–77. 10.1016/j.cell.2006.04.044 16839877

[phy214461-bib-0022] Hotamisligil, G. S. , Shargill, N. S. , & Spiegelman, B. M. (1993). Adipose expression of tumor necrosis factor‐alpha: Direct role in obesity‐linked insulin resistance. Science, 259(5091), 87–91. 10.1126/science.7678183 7678183

[phy214461-bib-0023] Kanda, H. , Tateya, S. , Tamori, Y. , Kotani, K. , Hiasa, K. , Kitazawa, R. , … Kasuga, M. (2006). MCP‐1 contributes to macrophage infiltration into adipose tissue, insulin resistance, and hepatic steatosis in obesity. Journal of Clinical Investigation, 116(6), 1494–1505. 10.1172/jci26498 16691291 PMC1459069

[phy214461-bib-0024] Kirkland, J. L. , Tchkonia, T. , Pirtskhalava, T. , Han, J. , & Karagiannides, I. (2002). Adipogenesis and aging: Does aging make fat go MAD? Experimental Gerontology, 37(6), 757–767. 10.1016/s0531-5565(02)00014-1 12175476

[phy214461-bib-0025] Kong, C. M. , Lee, X. W. , & Wang, X. (2013). Telomere shortening in human diseases. FEBS Journal, 280(14), 3180–3193. 10.1111/febs.12326 23647631

[phy214461-bib-0026] Liguori, I. , Russo, G. , Curcio, F. , Bulli, G. , Aran, L. , Della‐Morte, D. , … Abete, P. (2018). Oxidative stress, aging, and diseases. Clinical Interventions in Aging, 13, 757–772. 10.2147/cia.s158513 29731617 PMC5927356

[phy214461-bib-0027] Lindstrom, J. , & Tuomilehto, J. (2003). The diabetes risk score: A practical tool to predict type 2 diabetes risk. Diabetes Care, 26(3), 725–731. 10.2337/diacare.26.3.725 12610029

[phy214461-bib-0028] Liu, Y. , Bloom, S. I. , & Donato, A. J. (2019). The role of senescence, telomere dysfunction and shelterin in vascular aging. Microcirculation, 26(2), e12487 10.1111/micc.12487 29924435 PMC7135943

[phy214461-bib-0029] Liu, Z. , Patil, I. Y. , Jiang, T. , Sancheti, H. , Walsh, J. P. , Stiles, B. L. , … Cadenas, E. (2015). High‐fat diet induces hepatic insulin resistance and impairment of synaptic plasticity. PLoS ONE, 10(5), e0128274 10.1371/journal.pone.0128274 26023930 PMC4449222

[phy214461-bib-0030] Ludlow, A. T. , Gratidao, L. , Ludlow, L. W. , Spangenburg, E. E. , & Roth, S. M. (2017). Acute exercise activates p38 MAPK and increases the expression of telomere‐protective genes in cardiac muscle. Experimental Physiology, 102(4), 397–410. 10.1113/ep086189 28166612 PMC5378631

[phy214461-bib-0031] Ludlow, A. T. , Witkowski, S. , Marshall, M. R. , Wang, J. , Lima, L. C. J. , Guth, L. M. , … Roth, S. M. (2012). Chronic exercise modifies age‐related telomere dynamics in a tissue‐specific fashion. Journals of Gerontology: Series A, Biological Sciences and Medical Sciences, 67(9), 911–926. 10.1093/gerona/gls002 PMC3436090 22389464

[phy214461-bib-0032] Lumeng, C. N. , Liu, J. , Geletka, L. , Delaney, C. , Delproposto, J. , Desai, A. , … Yung, R. L. (2011). Aging is associated with an increase in T cells and inflammatory macrophages in visceral adipose tissue. The Journal of Immunology, 187(12), 6208–6216. 10.4049/jimmunol.1102188 22075699 PMC3237772

[phy214461-bib-0033] Mao, Z. , Seluanov, A. , Jiang, Y. , & Gorbunova, V. (2007). TRF2 is required for repair of nontelomeric DNA double‐strand breaks by homologous recombination. Proceedings of the National Academy of Sciences, 104(32), 13068–13073. 10.1073/pnas.0702410104 PMC1941808 17670947

[phy214461-bib-0034] Marseglia, L. , Manti, S. , D’Angelo, G. , Nicotera, A. , Parisi, E. , Di Rosa, G. , … Arrigo, T. (2014). Oxidative stress in obesity: A critical component in human diseases. International Journal of Molecular Sciences, 16(1), 378–400. 10.3390/ijms16010378 25548896 PMC4307252

[phy214461-bib-0035] Martinez, P. , Ferrara‐Romeo, I. , Flores, J. M. , & Blasco, M. A. (2014). Essential role for the TRF2 telomere protein in adult skin homeostasis. Aging Cell, 13(4), 656–668. 10.1111/acel.12221 24725274 PMC4326939

[phy214461-bib-0036] Matsutani, N. , Yokozaki, H. , Tahara, E. , Tahara, H. , Kuniyasu, H. , Haruma, K. , … Tahara, E. (2001). Expression of telomeric repeat binding factor 1 and 2 and TRF1‐interacting nuclear protein 2 in human gastric carcinomas. International Journal of Oncology, 19(3), 507–512. 10.3892/ijo.19.3.507 11494028

[phy214461-bib-0037] Mau, T. , & Yung, R. (2018). Adipose tissue inflammation in aging. Experimental Gerontology, 105, 27–31. 10.1016/j.exger.2017.10.014 29054535 PMC5869077

[phy214461-bib-0038] Minamino, T. , Orimo, M. , Shimizu, I. , Kunieda, T. , Yokoyama, M. , Ito, T. , … Komuro, I. (2009). A crucial role for adipose tissue p53 in the regulation of insulin resistance. Nature Medicine, 15(9), 1082–1087. 10.1038/nm.2014 19718037

[phy214461-bib-0039] Morgan, R. G. , Ives, S. J. , Lesniewski, L. A. , Cawthon, R. M. , Andtbacka, R. H. I. , Noyes, R. D. , … Donato, A. J. (2013). Age‐related telomere uncapping is associated with cellular senescence and inflammation independent of telomere shortening in human arteries. American Journal of Physiology‐Heart and Circulatory Physiology, 305(2), H251–H258. 10.1152/ajpheart.00197.2013 23666675 PMC3726958

[phy214461-bib-0040] Morgan, R. G. , Ives, S. J. , Walker, A. E. , Cawthon, R. M. , Andtbacka, R. H. I. , Noyes, D. , … Donato, A. J. (2014). Role of arterial telomere dysfunction in hypertension: Relative contributions of telomere shortening and telomere uncapping. Journal of Hypertension, 32(6), 1293–1299. 10.1097/hjh.0000000000000157 24686009 PMC4198301

[phy214461-bib-0041] Morgan, R. G. , Walker, A. E. , Trott, D. W. , Machin, D. R. , Henson, G. D. , Reihl, K. D. , … Donato, A. J. (2019). Induced Trf2 deletion leads to aging vascular phenotype in mice associated with arterial telomere uncapping, senescence signaling, and oxidative stress. Journal of Molecular and Cellular Cardiology, 127, 74–82. 10.1016/j.yjmcc.2018.11.014 30502348 PMC7216296

[phy214461-bib-0042] Munoz, P. , Blanco, R. , Flores, J. M. , & Blasco, M. A. (2005). XPF nuclease‐dependent telomere loss and increased DNA damage in mice overexpressing TRF2 result in premature aging and cancer. Nature Genetics, 37(10), 1063–1071. 10.1038/ng1633 16142233

[phy214461-bib-0043] Oh, B. K. , Kim, Y. J. , Park, C. , & Park, Y. N. (2005). Up‐regulation of telomere‐binding proteins, TRF1, TRF2, and TIN2 is related to telomere shortening during human multistep hepatocarcinogenesis. American Journal of Pathology, 166(1), 73–80. 10.1016/s0002-9440(10)62233-x 15632001 PMC1602303

[phy214461-bib-0044] Oh, Y. S. , Seo, E. H. , Lee, Y. S. , Cho, S. C. , Jung, H. S. , Park, S. C. , & Jun, H. S. (2016). Increase of calcium sensing receptor expression is related to compensatory insulin secretion during aging in mice. PLoS ONE, 11(7), e0159689 10.1371/journal.pone.0159689 27441644 PMC4956240

[phy214461-bib-0045] Palmer, A. K. , Xu, M. , Zhu, Y. I. , Pirtskhalava, T. , Weivoda, M. M. , Hachfeld, C. M. , … Kirkland, J. L. (2019). Targeting senescent cells alleviates obesity‐induced metabolic dysfunction. Aging Cell, 18(3), e12950 10.1111/acel.12950 30907060 PMC6516193

[phy214461-bib-0046] Prowse, K. R. , & Greider, C. W. (1995). Developmental and tissue‐specific regulation of mouse telomerase and telomere length. Proceedings of the National Academy of Sciences, 92(11), 4818–4822. 10.1073/pnas.92.11.4818 PMC41798 7761406

[phy214461-bib-0047] Reynolds, T. H. , Dalton, A. , Calzini, L. , Tuluca, A. , Hoyte, D. , & Ives, S. J. (2019). The impact of age and sex on body composition and glucose sensitivity in C57BL/6J mice. Physiological Reports, 7(3), e13995 10.14814/phy2.13995 30706674 PMC6356156

[phy214461-bib-0048] Sfeir, A. , Kosiyatrakul, S. T. , Hockemeyer, D. , MacRae, S. L. , Karlseder, J. , Schildkraut, C. L. , & de Lange, T. (2009). Mammalian telomeres resemble fragile sites and require TRF1 for efficient replication. Cell, 138(1), 90–103. 10.1016/j.cell.2009.06.021 19596237 PMC2723738

[phy214461-bib-0049] Shammas, M. A. (2011). Telomeres, lifestyle, cancer, and aging. Current Opinion in Clinical Nutrition and Metabolic Care, 14(1), 28–34. 10.1097/MCO.0b013e32834121b1 21102320 PMC3370421

[phy214461-bib-0050] Sowers, J. R. (2003). Obesity as a cardiovascular risk factor. American Journal of Medicine, 115(Suppl 8A), 37s–41s. 10.1016/j.amjmed.2003.08.012 14678864

[phy214461-bib-0051] Sun, K. , Kusminski, C. M. , & Scherer, P. E. (2011). Adipose tissue remodeling and obesity. Journal of Clinical Investigation, 121(6), 2094–2101. 10.1172/jci45887 21633177 PMC3104761

[phy214461-bib-0052] Takai, H. , Smogorzewska, A. , & de Lange, T. (2003). DNA damage foci at dysfunctional telomeres. Current Biology, 13(17), 1549–1556. 10.1016/s0960-9822(03)00542-6 12956959

[phy214461-bib-0053] Trayhurn, P. , & Wood, I. S. (2004). Adipokines: Inflammation and the pleiotropic role of white adipose tissue. British Journal of Nutrition, 92(3), 347–355. 10.1079/bjn20041213 15469638

[phy214461-bib-0014] U.S. Cancer Statistics Working Group . (2013). United States Cancer Statistics: 1999–2009 incidence and mortality web‐based report. Atlanta, GA: U.S. Department of Health and Human Services, Centers for Disease Control and Prevention and National Cancer Institute Retrieved from www.cdc.gov/uscs.

[phy214461-bib-0054] van der Heijden, R. A. , Bijzet, J. , Meijers, W. C. , Yakala, G. K. , Kleemann, R. , Nguyen, T. Q. , … Heeringa, P. (2015). Obesity‐induced chronic inflammation in high fat diet challenged C57BL/6J mice is associated with acceleration of age‐dependent renal amyloidosis. Scientific Reports, 5, 16474 10.1038/srep16474 26563579 PMC4643235

[phy214461-bib-0055] van Steensel, B. , Smogorzewska, A. , & de Lange, T. (1998). TRF2 protects human telomeres from end‐to‐end fusions. Cell, 92(3), 401–413. 10.1016/s0092-8674(00)80932-0 9476899

[phy214461-bib-0056] Vergoni, B. , Cornejo, P.‐J. , Gilleron, J. , Djedaini, M. , Ceppo, F. , Jacquel, A. , … Cormont, M. (2016). DNA damage and the activation of the p53 pathway mediate alterations in metabolic and secretory functions of adipocytes. Diabetes, 65(10), 3062–3074. 10.2337/db16-0014 27388216

[phy214461-bib-0057] von Zglinicki, T. (2002). Oxidative stress shortens telomeres. Trends in Biochemical Sciences, 27(7), 339–344. 10.1016/s0968-0004(02)02110-2 12114022

[phy214461-bib-0058] Walker, A. E. , Morgan, R. G. , Ives, S. J. , Cawthon, R. M. , Andtbacka, R. H. I. , Noyes, D. , … Donato, A. J. (2016). Age‐related arterial telomere uncapping and senescence is greater in women compared with men. Experimental Gerontology, 73, 65–71. 10.1016/j.exger.2015.11.009 26602606 PMC4792184

[phy214461-bib-0059] Wang, Z. , Rhee, D. B. , Lu, J. , Bohr, C. T. , Zhou, F. , Vallabhaneni, H. , … Liu, Y. (2010). Characterization of oxidative guanine damage and repair in mammalian telomeres. PLoS Genetics, 6(5), e1000951 10.1371/journal.pgen.1000951 20485567 PMC2869316

[phy214461-bib-0060] Yousefzadeh, M. J. , Zhao, J. , Bukata, C. , Wade, E. A. , McGowan, S. J. , Angelini, L. A. , … Niedernhofer, L. J. (2020). Tissue specificity of senescent cell accumulation during physiologic and accelerated aging of mice. Aging Cell, 19(3), e13094 10.1111/acel.13094 31981461 PMC7059165

[phy214461-bib-0061] Zhang, J. , Rane, G. , Dai, X. , Shanmugam, M. K. , Arfuso, F. , Samy, R. P. , … Sethi, G. (2016). Ageing and the telomere connection: An intimate relationship with inflammation. Ageing Research Reviews, 25, 55–69. 10.1016/j.arr.2015.11.006 26616852

[phy214461-bib-0062] Zhang, L. E. , Ebenezer, P. J. , Dasuri, K. , Fernandez‐Kim, S. O. , Francis, J. , Mariappan, N. , … Keller, J. N. (2011). Aging is associated with hypoxia and oxidative stress in adipose tissue: Implications for adipose function. American Journal of Physiology‐Endocrinology and Metabolism, 301(4), E599–E607. 10.1152/ajpendo.00059.2011 21586698 PMC3275102

